# Supplementary material for: Time-series transcriptome analysis identified differentially expressed genes in broiler chicken infected with mixed Eimeria species
Source: Front Genet. 2022 Aug 8;13:886781. doi: 10.3389/fgene.2022.886781 (PMC9393255; doi:10.3389/fgene.2022.886781)
Supplement: Supplementary file 2 [file DataSheet1.ZIP › 7dpi_KEGG.Gsea.1625115961950/KEGG_TYROSINE_METABOLISM.html]

Details for gene set KEGG\_TYROSINE\_METABOLISM[GSEA]

|  || Dataset | TMM\_7dpi\_gct\_format\_7dpi\_gct\_format.Class\_4dpi.cls #PC\_versus\_NC.Class\_4dpi.cls #PC\_versus\_NC\_repos |
| Phenotype | Class\_4dpi.cls#PC\_versus\_NC\_repos |
| Upregulated in class | 1 |
| GeneSet | KEGG\_TYROSINE\_METABOLISM |
| Enrichment Score (ES) | 0.3773227 |
| Normalized Enrichment Score (NES) | 1.3556322 |
| Nominal p-value | 0.09312639 |
| FDR q-value | 0.27652365 |
| FWER p-Value | 0.9128 |
Table: GSEA Results Summary

  

Fig 1: Enrichment plot: KEGG\_TYROSINE\_METABOLISM      
 Profile of the Running ES Score & Positions of GeneSet Members on the Rank Ordered List

  

| SYMBOL | TITLE | RANK IN GENE LIST | RANK METRIC SCORE | RUNNING ES | CORE ENRICHMENT || 1 | GSTZ1 | na | 161 | 1.120 | 0.0655 | Yes |
| 2 | GOT2 | na | 227 | 1.009 | 0.1311 | Yes |
| 3 | AOX1 | na | 282 | 0.956 | 0.1940 | Yes |
| 4 | MIF | na | 317 | 0.924 | 0.2562 | Yes |
| 5 | FAH | na | 546 | 0.712 | 0.2874 | Yes |
| 6 | GOT1 | na | 673 | 0.649 | 0.3226 | Yes |
| 7 | ADH1C | na | 914 | 0.551 | 0.3414 | Yes |
| 8 | METTL2B | na | 941 | 0.541 | 0.3773 | Yes |
| 9 | ALDH3B2 | na | 4510 | -0.051 | 0.0833 | No |
| 10 | IL4I1 | na | 4842 | -0.086 | 0.0617 | No |
| 11 | LCMT1 | na | 6398 | -0.243 | -0.0509 | No |
| 12 | AOC3 | na | 6496 | -0.253 | -0.0411 | No |
| 13 | ADH6 | na | 6644 | -0.269 | -0.0344 | No |
| 14 | MAOA | na | 6874 | -0.294 | -0.0328 | No |
| 15 | LCMT2 | na | 6916 | -0.299 | -0.0152 | No |
| 16 | METTL6 | na | 7400 | -0.352 | -0.0307 | No |
| 17 | HEMK1 | na | 9012 | -0.550 | -0.1263 | No |
| 18 | TRMT11 | na | 9330 | -0.603 | -0.1103 | No |
| 19 | DDC | na | 9496 | -0.631 | -0.0796 | No |
| 20 | ADH4 | na | 9849 | -0.682 | -0.0609 | No |
| 21 | COMT | na | 10088 | -0.726 | -0.0296 | No |
| 22 | MAOB | na | 11115 | -0.968 | -0.0470 | No |
| 23 | ALDH1A3 | na | 11961 | -1.725 | 0.0041 | No |
Table: GSEA details [plain text format]

  

Fig 2: KEGG\_TYROSINE\_METABOLISM      
 Blue-Pink O' Gram in the Space of the Analyzed GeneSet

  

Fig 3: KEGG\_TYROSINE\_METABOLISM: Random ES distribution      
 Gene set null distribution of ES for **KEGG\_TYROSINE\_METABOLISM**
